# Rare variants in genes related to inborn errors of immunity in patients with rheumatoid arthritis and secondary immunodeficiency

**DOI:** 10.1136/rmdopen-2026-007046

**Published:** 2026-07-03

**Authors:** Faranaz Atschekzei, Natalia Dubrowinskaja, Manfred Anim, Doris Steinemann, Sandra von Hardenberg, Winfried Hofmann, Robert Geffers, Torsten Witte, Georgios Sogkas

**Affiliations:** 1 Department of Rheumatology and Immunology, Hannover Medical School, Hannover, Germany; 2 Hannover Medical School, Cluster of Excellence RESIST (EXC 2155), Hanover, Germany; 3 Department of Human Genetics, Hannover Medical School, Hanover, Germany; 4 Biomedical Research in Endstage and Obstructive Lung Disease Hannover (BREATH), German Center for Lung Research (DZL), Hannover, Germany; 5 Helmholtz Centre for Infection Research, Braunschweig, Germany

**Keywords:** Arthritis, Rheumatoid, Polymorphism, Genetic, DMARD

## Abstract

**Objectives:**

Treatment of rheumatoid arthritis (RA) relies on immunomodulatory drugs that can compromise immunity, inducing secondary immunodeficiency (SID). However, features of SID, such as hypogammaglobulinaemia and severe/recurrent infections, occur in only a minority of patients, suggesting a potential underlying genetic predisposition. Given the expanding spectrum of genes implicated in inborn errors of immunity (IEIs) and the fact that IEIs can present with rheumatic manifestations, we hypothesised that a subset of patients with RA with SID harbour IEI-related variants.

**Methods:**

We screened 701 patients with RA for SID, defined as persistent hypogammaglobulinaemia or susceptibility to infections requiring prophylactic anti-infective therapy. Patients with SID underwent targeted whole-exome sequencing to identify rare variants in IEI-associated genes.

**Results:**

Among 701 evaluated patients, 70 (10%) had SID. SID was more frequently observed in patients with earlier onset of RA, seronegative disease and in those receiving rituximab therapy. Genetic analysis identified 17 putatively pathogenic variants in 15 of 70 patients with SID (21.4%). All variants were monoallelic, with 7 of 17 (41.2%) affecting genes involved in canonical NF-κB signalling. Two patients (3.1%) harboured variants previously reported as pathogenic.

**Conclusions:**

Although rare, underlying IEIs can account for immunodeficiency in patients with RA. Identifying patients with IEIs among those with RA may have important implications for disease management, as it can guide the selection of immunomodulatory therapy and help prevent infectious complications. Furthermore, it may refine our interpretation of drug safety, particularly in cases of unusual infections that may instead be attributable, rather, to an underlying germline defect.

**WHAT IS ALREADY KNOWN ON THIS TOPIC**

**WHAT THIS STUDY ADDS**

- IEIs such as NF-κB insufficiency and signal transducer and activator of transcription 3-gain-of-function are rare but exist among patients with RA and immunodeficiency perceived as secondary to disease-modifying antirheumatic drugs, that is, secondary immunodeficiency (SID) IEIs in patients with RA with SID are more common among patients with a suggestive family history.

**HOW THIS STUDY MIGHT AFFECT RESEARCH, PRACTICE OR POLICY**

- Genetic testing for an underlying IEI should be considered in patients with RA with SID. Larger studies are needed to evaluate the features predicting an underlying IEI in patients with RA with SID.

## Introduction

 Rheumatoid arthritis (RA) is a chronic, systemic immune-mediated disease characterised by inflammatory arthritis and a pronounced heterogeneity in disease course, severity and extra-articular manifestations.^1^ At diagnosis of RA, treatment guidelines recommend initiation of methotrexate (MTX) or an alternative conventional synthetic disease-modifying antirheumatic drug (csDMARD). In case of failure or inadequate response to csDMARDs, biologic or targeted synthetic DMARDs (bDMARDs or tsDMARDs, respectively) can be employed to achieve adequate disease control.^2^ Although DMARDs can exert immunosuppressive effects, only a slight increase in the risk of infections has been suggested by registry studies and large meta-analyses.^3^,^6^ However, certain DMARDs can increase the risk of specific infections, such as tuberculosis with TNF inhibitors or herpes zoster with Janus kinase inhibitors (JAKi),^4 7 8^ while glucocorticoids appear to increase the risk for infections in a dose-dependent manner.^9^ With the exception of rituximab (RTX)-treated patients, among whom approximately 20% develop hypogammaglobulinaemia, this condition remains uncommon in patients treated with alternative DMARDs.^10 11^ Furthermore, cases of clinically significant secondary hypogammaglobulinaemia, associated with recurrent infections, appear to be even less frequent.

Inborn errors of immunity (IEI) comprise a heterogeneous group of genetic disorders characterised by profound immune dysfunction.^12^ Besides impaired host defence manifesting as recurrent or severe infections, the phenotypic spectrum of IEI includes features of immune dysregulation, such as autoimmunity and autoinflammation; in a subgroup of IEI, often classified as disorders of immune dysregulation or autoinflammatory diseases, immune dysregulation constitutes the predominant clinical phenotype. The clinical presentation, especially of the latter two subgroups of IEI, can mimic systemic rheumatic diseases, characterised by inflammatory arthritis, vasculitis, interstitial lung disease (ILD) and cutaneous manifestations like eczema.^13^ Distinguishing IEI-associated rheumatic manifestations from polygenic rheumatic diseases has important therapeutic implications, as it may justify avoiding broadly immunosuppressive agents and instead favouring targeted, mechanism-based treatments. Examples include the use of JAK inhibitors in patients with signal transducer and activator of Transcription 3 (STAT3) gain-of-function syndrome, leniolisib in patients with activated phosphoinositide 3-kinase delta syndrome (APDS) and abatacept in patients with cytotoxic T-lymphocyte-associated protein 4 (CTLA-4) insufficiency or lipopolysaccharide-responsive beige-like anchor protein (LRBA) deficiency.^14^,^16^

In a previous cohort of patients with diverse rheumatic diseases and persistent secondary immunodeficiency (SID), we identified genetic variants in genes associated with IEI by means of targeted next-generation sequencing (NGS).^17^ Notably, a subset of tested patients harboured deleterious variants consistent with the diagnosis of a monogenic IEI. In addition, several patients with well-characterised rheumatic diseases and increased susceptibility to infections were later diagnosed with an IEI through genetic testing, whereas their infectious complications had previously been attributed to DMARD therapy.^18 19^ These findings provided evidence for the presence of monogenic IEI among patients with rheumatic disorders and the genetic aetiology of immunodeficiency in this subset of patients. Here, given the continuously expanding spectrum of genes implicated in IEI, we employed whole-exome sequencing (WES) to identify rare genetic variants in IEI-associated genes in a cohort of patients with RA who developed immunodeficiency following the initiation of DMARD therapy.

## Patients and methods

### Study cohort

This single-centre study included all patients with RA visiting our Rheumatology clinics between December 2022 and November 2024 (N=732). The diagnosis of RA was established according to the 2010 American College of Rheumatology/European League Against Rheumatism (ACR/EULAR) classification criteria.^20^ Patients were followed at regular intervals, with study visits scheduled approximately every 3–6 months. At each visit, serum Ig concentrations were assessed. The reference range for serum IgG levels in adults is 7–16 g/L. SID was defined by the presence of persistent secondary hypogammaglobulinaemia and/or a pathological susceptibility to infections. Persistent secondary hypogammaglobulinaemia was defined as continuously reduced serum IgG levels (<7 g/L) documented for at least 12 months prior to study inclusion and persisting throughout the follow-up period, with onset occurring after initiation of immunomodulatory therapy.^21^ Pathological susceptibility to infections was defined as repeated infectious episodes occurring after initiation of immunomodulatory therapy, requiring repeated anti-infective treatment and resulting in the initiation of immunoglobulin replacement therapy or prophylactic antimicrobial treatment, including antibiotics, antiviral agents and antifungal agents.

### Whole-exome sequencing

Blood samples were collected at the clinics of the Department of Rheumatology and Immunology of the Hannover University Hospital. Genomic DNA (gDNA) was isolated from peripheral whole blood using QIAamp DNA Blood Midi Kit, according to the manufacturer’s protocol (Qiagen). WES was performed on gDNA samples from patients fulfilling the aforementioned criteria of SID. Concentration and quality of the purified gDNA were determined with an Agilent Technologies 2100 Bioanalyzer. The DNA sequencing library consisted of 100 ng fragmented gDNA and was generated with Agilent SureSelectXT Reagent Kits v5 UTR (70 Mb) according to the manufacturer’s protocols (Illumina, San Diego, California, USA). Libraries were sequenced on an Illumina HiSeq2500 platform using TruSeq SBS Kit v3-HS (200 cycles, paired-end run) with an average of 12.5×10^6^ reads per single exome (mean coverage: 50×). Generated FastQ files were processed by the ‘analyse’-NGS-pipeline of megSAP (Medical Genetics Sequence Analysis Pipelines, https://github.com/imgag/megSAP/), including the alignment to the human reference genome (Genome Reference Consortium Human Build 38 (GRCh38/hg38)), variant calling and annotation. Filtering of the resulting variant data was done using the software GSvar (https://github.com/imgag/ngs-bits/) and visualisation with the Integrative Genomics Viewer.^22^

Variants were filtered based on their presence in exonic and splice site regions of genes associated with IEI causing immunodeficiency, as classified by the International Union of Immunological Societies expert committee (online supplemental text).^12^ Only variants with a minor allele frequency of ≤1% in the 1000 Genomes Project or the Genome Aggregation Database (gnomAD) were included.^23^ Intronic variants were excluded from further analysis. Missense variants were evaluated using the ‘Sorting Intolerant From Tolerant’ (SIFT), Polymorphism Phenotyping v2 (PolyPhen2), Mutation Taster and GeneSplicer.^24^,^27^ Additional classification relied on datasets including ClinVar, HGMD, combined annotation-dependent depletion (CADD) score (deleteriousness threshold >20) and phyloP (deleterious threshold >1.6).^28^,^31^ Variant classification adhered to the standards and guidelines of the American College of Medical Genetics and Genomics (ACMG) and the Association for Molecular Pathology (AMP).^32^

### Statistical analysis

Descriptive statistics are reported as median and IQR in case of continuous variables and as counts and percentages for dichotomous variables. Categorical variables were compared by Fisher’s exact test. Non-categorical variables were compared with the Mann-Whitney U test. The aforementioned tests were performed using GraphPad Prism V.10 (GraphPad, La Jolla, USA). A multivariable logistic regression analysis was performed with EasyMedStat V.4.4 (Levallois-Perret, France) to identify independent factors associated with SID. ORs with 95% CI were calculated.

## Results

### Comparison of patients with RA with and without SID

Among 732 patients with RA screened for SID, 29 were excluded, including 25 with inadequate data and 4 with an alternative non-DMARD-related aetiology of immunodeficiency. Two additional patients who fulfilled the SID criteria were excluded because they had previously been diagnosed with either STAT3 gain-of-function disease or NF-κB1 insufficiency (patient identification numbers 33 and 59).^17^ Overall, 701 patients were evaluated, of whom 70 (10%) were diagnosed with SID. Comparison of patients with and without SID revealed that while patient age between those groups was comparable (table 1), patients in the SID group were diagnosed with RA at a significantly younger age (median 43.7 vs 50.4 years, p=0.0052), suggesting a longer cumulative treatment exposure. Interestingly, patients with SID exhibited a significantly lower seropositivity rate compared with the non-SID group (52.9% vs 75.3%; OR: 0.37; 95% CI 0.22 to 0.61; p=0.0002). No significant differences were observed regarding sex or extra-articular manifestations, including ILD and vasculitis. Comparison of anti-inflammatory treatment revealed no differences in the use of csDMARDs between patients with and without SID. With respect to bDMARDs, RTX treatment was significantly more common in patients with SID (37.1% vs 23.5%; OR: 1.93; 95% CI 1.14 to 3.2; p=0.0188). The use of other bDMARDs and tsDMARDs (ie, JAKi) was similar between the two groups. With respect to glucocorticoid therapy, low-dose regimens (prednisolone-equivalent dose <10 mg/day) were significantly less frequent in patients with SID than in those without (7.1% vs 36.6%; OR: 0.13; 95% CI 0.06 to 0.32; p<0.0001), possibly reflecting treating rheumatologists’ efforts to minimise infection risk. In order to adjust for potential confounders for SID, including DMARD treatment, demographic and clinical variables, we performed a multivariable logistic regression analysis (online supplemental table 1). This confirmed the findings of the univariate analysis, showing that RTX treatment and younger age at diagnosis of RS were independently associated with SID, whereas the RA type, that is, seropositive disease, was associated with lower odds of SID.

**Table 1:** Patients’ characteristics and immunomodulatory regimens at study inclusion

Characteristic	w/o SID (N=631)	SID (N=70)	P value*
Median age (IQR)–years	62.6 (52.6–73.3)	61.6 (52.8–71.4)	0.9722
Age at diagnosis of RA (IQR)–years	50.4 (38.7–60.4)	43.7 (32–54.9)	0.0052**
Male sex–no (%)	168 (26.6)	14 (20)	0.2534
Seropositive–no (%)	475 (75.3)	37 (52.9)	0.0002***
IgA (IQR)–g/L	2.26 (1.55–3.19)	1.16 (0.76–1.79)	<0.0001****
IgG (IQR)–g/L	10.41 (8.59–12.46)	5.52 (4.78–6.5)	<0.0001****
IgM (IQR)–g/L	0.89 (0.6–0.89)	0.55 (0.31–1.13)	<0.0001****
Extra-articular manifestations			
Associated SjD	35 (5.5)	8 (11.4)	0.0637
ILD	33 (5.2)	3 (4.3)	>0.9999
Vasculitis	13 (2.1)	4 (5.7)	0.0798
csDMARD			
MTX-no (%)	259 (46.8)	29 (41.4)	>0.9999
LFN-no (%)	25 (4)	3 (4.3)	0.753
SSZ-no (%)	34 (5.4)	1 (1.4)	0.2421
HCQ-no (%)	75 (119)	6 (8.6)	0.554
bDMARD			
RTX-no (%)	148 (23.5)	26 (37.1)	0.0188*
TNFi-no (%)	119 (18.9)	8 (11.4)	0.1425
IL-6Ri-no (%)	29 (4.6)	3 (4.3)	>0.9999
Abatacept-no (%)	26 (4.1)	5 (7.1)	0.2239
tsDMARD			
JAKi-no (%)	53 (8.4)	6 (8.6)	>0.9999
Glucocorticoids			
GC <10 mg-no (%)	231 (36.6)	5 (7.1)	<0.0001****
GC ≥10 mg-no (%)	4 (0.6)	0 (0)	>0.9999

* p<0.05, **p<0.01, ***p<0.001, ****p<0.0001.

bDMARD, biological disease-modifying antirheumatic drug; csDMARDs, conventional synthetic DMARDs; GC, glucocorticoids; HCQ, hydroxychloroquine; ILD, interstitial lung disease; IL-6Ri, interleukin 6six receptor inhibitor; JAKi, Janus kinase inhibitors; LFN, leflunomide; MTX, methotrexate; N, total number; no, number; RA, rheumatoid arthritis; RTX, rituximab; SID, secondary immunodeficiency; SjD, Sjögren’s disease; SSZ, sulfasalazine; TNFi, tumour necrosis factor inhibitor; tsDMARDs, targeted synthetic DMARDs.

### Infections and management of SID in RA

**Figure 1:**
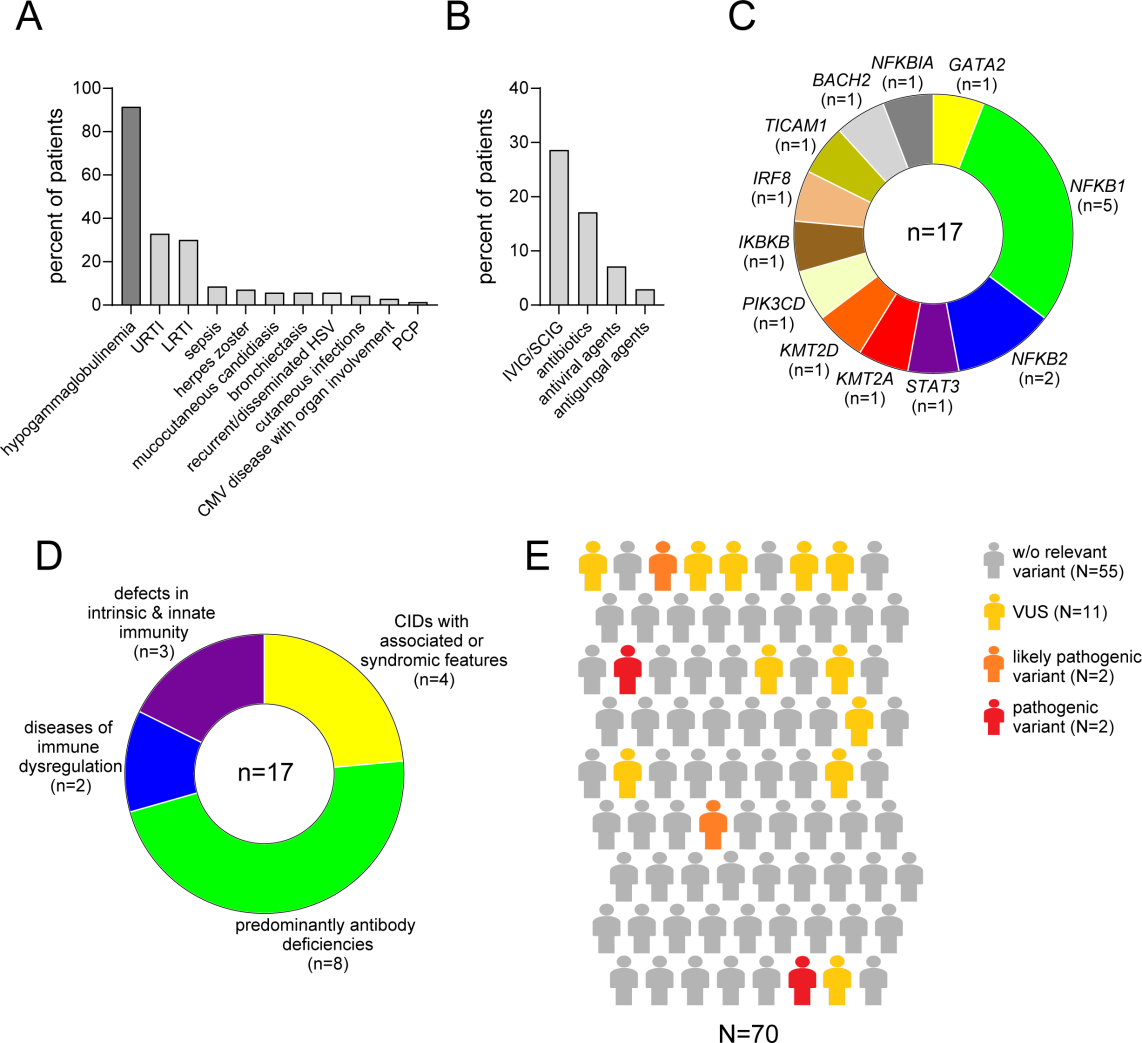
Clinical features and genetic findings in patients with rheumatoid arthritis (RA) with secondary immunodeficiency (SID). Percentage of patients with persistent hypogammaglobulinaemia and infectious complications (**A**), including upper respiratory tract infections (URTI), lower respiratory tract infections (LRTI), recurrent/disseminated Herpes simplex virus (HSV) infections and *Pneumocystis jirovecii* pneumonia (PCP). Further, quotes of patients with prophylactic anti-infective treatment, including immunoglobulin replacement and prophylactic antibiotics, antiviral or antifungal agents (**B**). Summary of genes, whose variants (n=17) were detected in a cohort of patients with RA and SID (**C**) and related disorders according to the classification of inborn errors of immunity (**D**). Finally, variant classification according to the ACMG/AMP criteria, identified in tested patients with SID (N=70). ACMG/AMP, American College of Medical Genetics and Genomics/Association for Molecular Pathology; CIDs, combined immunodeficiencies; CMV, cytomegalovirus; SCIG, subcutaneous immunoglobulin; VUS, variants of uncertain significance.

A total of 64 of 70 patients with SID (91.4%) exhibited hypogammaglobulinaemia (figure 1A), of whom 31 (44.3%) were receiving neither prophylactic anti-infective therapy nor immunoglobulin replacement. Given the association between RTX exposure and hypogammaglobulinaemia,^10^ baseline immunoglobulin levels prior to RTX exposure were additionally evaluated. However, no significant differences compared with the remaining SID subgroup were observed (online supplemental figure 1). Six of 70 patients (8.6%) with SID did not exhibit hypogammaglobulinaemia, but had a history of recurrent infections that led to prophylactic anti-infective therapy. The most common infectious complications were recurrent upper respiratory tract infection (URTI) (22/70, 31.4%) followed by LRTI (21/70, 30%). Septic episodes were documented in 6 cases (8.5%). Viral infections included herpes zoster (5/70, 7.1%) or recurrent/disseminated mucocutaneous herpes simplex virus (HSV) reactivations (4/70, 5.7%). Serious opportunistic infections were identified in a small subset of patients. In particular, one patient had a history of herpes simplex encephalitis (HSE). Two patients had a history of cytomegalovirus colitis and one patient had previously suffered from *Pneumocystis jirovecii* pneumonia (PCP). The management of SID included immunoglobulin replacement therapy in 20 of 70 patients (28.6%), including 8 patients receiving subcutaneous immunoglobulin and prophylactic antibiotic treatment in 12 of 70 patients (17.1%). A smaller subset of patients with a history of viral or fungal infections received prophylactic antiviral therapy (5/70, 7.1%) or antifungal agents (2/70, 2.9%), respectively (figure 1B).

### Identification of rare variants in genes related to autosomal dominant IEIs in patients with RA with SID

WES was performed with a focus on identifying variants in established immunodeficiency-associated genes implicated in IEIs. Considering allele frequency and in silico deleteriousness prediction scores, including CADD, PolyPhen2 and SIFT, we identified a total of 17 rare, putatively pathogenic variants in genes linked to autosomal dominant IEIs in 15 of 70 sequenced patients (21.4%) (figure 1C, table 2). All those 15 patients fulfilled the criterion of secondary hypogammaglobulinaemia, while seven out of them were also on prophylactic anti-infective treatment, including immunoglobulin replacement. All identified variants were monoallelic. The majority of variants were located in genes associated with predominantly antibody deficiencies (8/17, 47.1%), namely *NFKB1*, *NFKB2* and *PIK3CD*, followed by genes related to combined immunodeficiencies (CIDs) with associated or syndromic features (4/17, 23.5%; *IKBKB*, *KMT2A*, *KMT2D* and *NFKBIA*), defects in intrinsic and innate immunity (3/17, 17.6%; *GATA2*, *IRF8* and *TICAM1*) and diseases of immune dysregulation (2/17, 11.8%; *BACH2* and *STAT3*) (figure 1D). According to ACMG/AMP criteria, most variants were classified as variants of uncertain significance (VUS) (13/17, 76.5%), whereas two variants were classified as likely pathogenic (11.8%) and two other variants as pathogenic (11.8%) (table 2).

**Table 2:** Monoallelic variants in genes associated with autosomal dominant IEIs, identified in patients with RA and SID

Pat. ID.	Gene	chr./position	HGVS nomenclature	gnomAD allele frequency	Reference SNP	CADD	PolyPhen	PolyPhen-pred.*	SIFT	SIFT-pred.†	ACMG/AMP classification
1	*GATA2*	3/128486063	NM_032638.5:c.817A>C (p.Lys273Gln)	0.0000	n.a.	28.0	0.86	D	0.01	D	VUS
3	*NFKB2*	10/102399656	NM_002502.5:c.1416_1417del (p.(Leu473AlafsTer32)	n.a.	n.a.	n.a.	n.a.	n.a.	n.a.	n.a.	LP
4	*NFKB1*	4/102616502	NM_003998.4:c.2818A>C (p.Thr940Pro)	0.0000	rs750315573	22.1	0.90	D	0.00	D	VUS
5	*NFKB1*	4/102566997	NM_003998.4:c.269A>T (p.Tyr90Phe)	0.0000	rs764399130	23.7	0.08	B	0.00	D	VUS
7	*NFKB1*	4/102616502	NM_003998.4:c.2818A>C (p.Thr940Pro)	0.0000	rs750315573	22.1	0.90	D	0.00	D	VUS
8	*NFKB1*	4/102616502	NM_003998.4:c.2818A>C (p.Thr940Pro)	0.0000	rs750315573	22.1	0.90	D	0.00	D	VUS
8	*NFKB2*	10/102401852	NM_002502.5:c.2401C>T (p.Arg801Cys)	0.0000	rs371174743	23.2	0.01	B	0.03	D	VUS
20	*STAT3*	17/42329430	NM_139276.3:c.1261G>A (p.Gly421Arg)	0.0000	rs869312888	29.8	0.76	P	0.00	D	P
24	*KMT2D*	12/49028051	NM_003482.4:c.14473C>T (p.Arg4825Trp)	0.0001	rs117904191	24.3	0.93	D	0.02	D	VUS
26	*IRF8*	16/85903061	NM_002163.4:c.46G>A (p.Glu16Lys)	0.0000	rs1347366121	32.0	0.83	P	0.13	T	VUS
35	*KMT2A*	11/118504644	NM_005933.4:c.8743A>C (p.Ile2915Leu)	n.a.	n.a.	23.7	0.08	B	0.01	D	VUS
38‡	*NFKB1*	4/102616477	NM_003998.4:c.2793G>C (p.Glu931Asp)	n.a.	n.a.	24.8	0.98	D	0.00	D	VUS
38	*IKBKB*	8/42316720	NM_001556.3:c.941T>C (p.Ile314Thr)	0.0002	rs200044839	24.8	0.37	P	0.00	D	VUS
44‡	*PIK3CD*	1/9724883	NM_005026.3:c.2944C>T (p.Arg982Trp)	0.0000	rs778194087	28.5	0.93	D	0.02	D	VUS
49	*TICAM1*	19/4816708	NM_182919.3:c.1517_1670del (p.Leu506ProfsTer10)	n.a.	n.a.	n.a.	n.a.	n.a.	n.a.	n.a.	LP
68‡	*NFKBIA*	14/35402618	NM_020529.3:c.682C>T (p.Gln228Ter)	n.a.	n.a.	43.0	n.a.	n.a.	n.a.	n.a.	P
69	*BACH2*	6/89950760	NM_021813.3:c.1346C>T (p.Ser449Phe)	0.0000	rs988017377	24.4	0.47	P	0.05	T	VUS

* Classified as B (benign), P (possibly damaging) or D (probably damaging).

† Classifed as D (deleterious) or T (tolerated).

‡ These patients were tested previously also with targeted NGS (see Elsayed *et al*^18^).

ACMG, American College of Medical Genetics and Genomics; AD, autosomal dominant; AMP, Association for Molecular Pathology; AR, autosomal recessive; chr, chromosome; D, damaging; F, female; freq., frequency; HGVS, Human Genome Variation Society; IEI, inborn errors of immunity; LP, likely pathogenic; n.a., not available; P, pathogenic; Pat. ID, patient identification number; pred., prediction; RA, rheumatoid arthritis; ref.seq., reference sequence; SID, secondary immunodeficiency; SIFT, sorting intolerance from tolerance; SNP, single nucleotide polymorphism; t, tolerated; VUS, variant of uncertain significance.

Notably, 5 of the 17 variants (29.4%) were localised in *NFKB1*, encoding the p105 subunit of NF-κB1, which is commonly associated with CVID, though *NFKB1*-associated disease is marked by phenotypic and immunological heterogeneity across both haploinsufficient and missense variants.^33 34^ All five variants were missense and were identified in five patients with SID, including hypogammaglobulinaemia and recurrent respiratory tract infection leading to prophylactic antibiotics or immunoglobulin replacement in two cases (table 3, online supplemental table 2). Further, four of these patients displayed RA complexed with extraarticular features, including vasculitis, recurrent scleritis and rheumatoid nodules. Among identified *NFKB1* variants, the c.2842A>C; p.(Thr948Pro) variant was identified in three unrelated patients, who all displayed hypogammaglobulinaemia. Despite its recurrence, this variant remains extremely rare in population databases (allele frequency in the Genome Aggregation Database (gnomAD): 0.00001115) and has not been identified previously in our institutional genetics database. Although the recurrent detection in patients with overlapping phenotypic features raises the possibility of clinical relevance, no segregation or functional analyses were performed. Therefore, the variant remains classified as a VUS. A patient with an *NFKB1* variant also harboured a variant in *IKBKB. IKBKB* encodes the Inhibitor of κB kinase β (IKKβ), a kinase essential for canonical NF-κB signalling and immune cell activation.^35^ Monoallelic gain-of-function variants in *IKBKB* have been reported to cause severe CID. Additionally, a missense variant in *NFKBIA*, predicted to result in a stop-gain, was recently confirmed to negatively affect canonical NF-κB activation, which suggested that it can cause immunodeficiency and autoinflammation by a mechanism shared with variants resulting in NF-κB1 insufficiency.^19^ Two patients were identified with variants in *NFKB2*, one of which had a predicted frameshift effect. Monoallelic variants in *NFKB2* have been reported to cause a CVID-like disorder characterised by autoimmunity.^36^ A single patient harboured a *STAT3* variant (c.1261G>A; p.(Gly421Arg)), previously reported to cause STAT3 gain-of-function, an IEI characterised by autoimmunity, lymphoproliferation and immunodeficiency.^37^ Similar to this case, the previously reported patient presented with polyarthritis. Variants in *KMT2A* and *KMT2D*, genes associated with Kabuki syndrome, were identified in two patients, neither of whom fulfilled the international consensus diagnostic criteria for Kabuki syndrome, as no characteristic facial dysmorphism or additional syndromic features were documented.^38^ However, the retrospective design of the study represents a limitation, as no dedicated dysmorphologic evaluation was performed following variant identification and subtle syndromic features may therefore not have been systematically assessed or documented. A variant in *TICAM1*, the gene encoding TRIF, Toll/interleukin-1 receptor-domain-containing adapter-inducing interferon-β, an adaptor involved in Toll-like receptor 3 (TLR3) signalling, was identified in a patient with recurrent HSV type 1 (HSV-1) reactivations, manifesting as labial and nasal herpes as well as herpes ophthalmicus, necessitating aciclovir prophylaxis. Biallelic as well as monoallelic *TICAM1* has been associated with HSE.^39^ In a patient with a history of urogenital tuberculosis who additionally developed RTX-associated hypogammaglobulinaemia and recurrent respiratory tract infections requiring cotrimoxazole prophylaxis, we identified an IRF8 variant. IRF8 encodes an interferon-regulated transcription factor involved in monocyte and dendritic-cell development as well as IL-12-dependent immunity.^40^ Biallelic as well as monoallelic variants have been reported to cause Mendelian susceptibility to mycobacterial disease (MSMD) through disruption of the IL-12/IFN-γ axis. However, as no further infectious manifestations typical of MSMD were documented and the mycobacterial infection may have been confounded by immunosuppressive therapy, the clinical relevance of this VUS remains unclear. In addition, we identified a rare missense variant in *PIK3CD* in a patient with a history of recurrent URTI, a *GATA2* variant in a patient with respiratory tract infections and cryptogenic liver cirrhosis, including pneumonia episodes and a *BACH2* variant in a patient with recurrent folliculitis and bronchitis. In patients harbouring these latter three variants, aside from hypogammaglobulinaemia and infections consistent with humoral immunodeficiency, no additional features typically associated with the respective gene-related disorders were observed—namely, lymphoproliferation for *PIK3CD* variants, haematologic malignancies or features of MonoMAC syndrome for *GATA2* variants or Tregopathy-related manifestations for *BACH2* variants.^41^,^43^ Despite the broad phenotypic variability reported for related disorders, including APDS, GATA2 deficiency and BACH2-related immunodeficiency and autoimmunity, the clinical relevance of VUS identified in the respective genes remains uncertain.

**Table 3:** Clinical data of patients with monoallelic variants in genes associated with autosomal dominant IEIs

Pat. ID	Gene	Age	Sex	Age at RA	Age at SID	Infections/immunoglobulin replacement, prophylactic anti-infective treatment	Other features	DMARD and GC at diagnosis of SID
1	*GATA2*	60.2	F	38	38.4	Recurrent URTI, pneumonia, sepsis/IVIG	Liver cirrhosis attributed to NSAID	LFN, prednisolone 13 mg/day
3	*NFKB2*	68.6	F	32.4	60	Recurrent URTI, pneumonia	n.d.	AZA, prednisolone 5 mg/day
4	*NFKB1*	69.4	M	62.9	65	Recurrent URTI, pneumonia/cotrimoxazole	Vasculitis	CYC, prednisolone 5 mg/day
5	*NFKB1*	69	M	61.1	62.9	n.d.	SjD, Raynaud’s phenomenon, recurrent episcleritis and scleritis, atypical optic neuritis, melanoma, NMSC	MTX, HCQ, prednisolone 5 mg/day
7	*NFKB1*	66.6	F	52.9	63.8	Recurrent URTI, bronchitis/SCIG	Temporal arteritis, angiomyxoid neoplasm of the left kidney	MTX, prednisolone 5 mg/day
8	*NFKB1*/*NFKB2*	67.9	F	65.3	65.8	n.d.	n.d.	MTX, prednisolone 20 mg/day
20	*STAT3*	36.8	F	28.3	29.2	Recurrent URTI/SCIG, azithromycin	Atopic dermatitis	MTX, prednisolone 10 mg/day
24	*KMT2D*	61	M	52.6	59.5	n.d.	ILD	MTX, prednisolone 10 mg/day
26	*IRF8*	80.8	F	41.4	70	Urogenital tuberculosis, recurrent erysipelas, pneumonia/cotrimoxazole	Multilocular CIS of the vagina	RTX
35	*KMT2A*	42.7	F	19.4	35.1	n.d.	n.d.	MTX, prednisolone 5 mg/day
38*	*NFKB1*/*IKBKB*	59.4	F	28	44	n.d.	Rheumatoid nodules	TCZ, prednisolone 5 mg/day
44*	*PIK3CD*	58.8	M	40.7	43.7	Recurrent URTI	n.d.	Etanercept, MTX
49	*TICAM1*	71.9	F	54.5	64.6	Recurrent URTI, recurrent HSV1 reactivation, including labial, nasal herpes and herpes ophthalmicus, *Clostridioides difficile* colitis/IVIG, aciclovir	Cylindrical bronchiectasis	Abatacept, MTX
68*	*NFKBIA*	60.3	M	25	53	n.d.	Dyshidrotic eczema, psoriasis	TCZ, prednisolone 5 mg/day
69	*BACH2*	27.3	M	24.9	25.4	Recurrent URTI, pneumonia/IVIG	n.d.	RTX

AZA, azathioprine; CIS, carcinoma in situ; CYC, cyclophosphamide; DMARD, disease-modifying antirheumatic drug; F, female; GC, glucocorticoids; HCQ, hydroxychloroquine; IEI, inborn errors of immunity; ILD, interstitial lung disease; LFN, leflunomide; MTX, methotrexate; n.d., not documented; NMSC, non-melanoma skin cancer; no., number; Pat. ID, patient identification number; RA, rheumatoid arthritis; RTX, rituximab; SCIG, subcutaneous immunoglobulin; SID, secondary immunodeficiency; SjD, Sjögren’s disease; URTI, upper respiratory tract infections.

### Comparison with previous targeted NGS data on patients with RA with persistent secondary hypogammaglobulinaemia

Targeted NGS panels are restricted to a selected group of genes and may therefore miss causative variants, while WES enables a more comprehensive evaluation of coding regions across IEI-related genes. In a previous study, we employed targeted NGS panels to identify IEI in a cohort of rheumatic patients with persistent secondary hypogammaglobulinaemia.^17^ To enable a direct comparison of the diagnostic yield between the current WES-based analysis and our previous study employing targeted NGS (see online supplemental text), we excluded patients with RA who had already been screened in the earlier mixed cohort of rheumatic patients, including those in whom variants potentially underlying IEI had been identified (marked with a ‘*’ in table 2). In addition, to enable comparison between both studies, we excluded patients without persistent secondary hypogammaglobulinaemia, as only patients with persistent secondary hypogammaglobulinaemia underwent genetic testing in the previous targeted NGS cohort.^17^ Despite the anticipated higher diagnostic yield of WES, the comparison of these two independent cohorts revealed similar proportions of patients harbouring variants in IEI-related genes, including comparable frequencies of pathogenic or likely pathogenic variants according to ACMG/AMP criteria (online supplemental table 1). This finding may be explained by the fact that the identified variants were commonly located in genes that were assessed in both the current and the previous study, that is, by both sequencing approaches.

Despite the relatively low number of patients with variants, the identification of IEI-associated variants in an independent RA cohort corroborates the finding of our previous study, while expanding the spectrum of genes associated with SID by the identification of variants in genes related to defects of intrinsic and innate immunity as well as CIDs with associated or syndromic features. The consistent identification of IEI-related variants across two distinct cohorts supports the notion that a subset of patients classified as having SID may, in fact, harbour an underlying genetic predisposition overlapping with IEIs.

### Exploratory analysis of clinical features in patients with RA with SID harbouring pathogenic or likely pathogenic IEI variants

In addition to hypogammaglobulinaemia and infectious complications, previous studies have suggested that manifestations of immune dysregulation may occur in patients with underlying IEIs presenting with rheumatic disease.^13^ Considering patients with RA identified with pathogenic or likely pathogenic variants, in the present or our previous study^17^ and comparing them with patients who remained without a putatively pathogenic variant (including a VUS) despite genetic testing by means of WES, we evaluated the likely differential frequency of features proposed as ‘red flags’ for an underlying IEI. Patients with pathogenic/likely pathogenic variants numerically more frequently had young-onset RA (<40 years), with 66.7% affected versus 29.1% in the subgroup without predicted pathogenic variants (p=0.0837), which, however, did not reach statistical significance (table 4). Comorbid autoimmune or immunologic manifestations, including associated Sjögren’s disease, ILD, vasculitis or other manifestations of immune dysregulation, were generally infrequent and their prevalence did not differ significantly between groups. Also, the rates of difficult-to-treat RA, evaluated according to the EULAR definition at the last follow-up visit, were comparable between the two groups.^44^ As expected, given the hereditary nature of IEIs, the most striking difference was observed for family history of autoimmunity or immunodeficiency: all patients with pathogenic or likely pathogenic variants (100%) had a first-degree relative affected by an autoimmune disease, chronic inflammatory disease and/or a primary immunodeficiency, compared with 25.5% in non-carriers (p=0.0007). The latter underscores the importance of systematically documenting family history, with the presence of first-degree relatives affected by immune-related disorders representing a key clinical indicator for initiating genetic testing for an underlying IEI.

**Table 4:** Evaluation of possible red flags suggestive of underlying IEI in patients with RA and SID

Characteristic	Pathogenic/likely pathogenic variant (N=6)	No variant (N=55)	OR	95% CI	P value*
Age at diagnosis of RA (IQR)–years	33.1 (27.6–58.1)	43.9 (34.5–53.5)	n.a.	n.a.	0.5461
Young-onset†–no (%)	4 (66.7)	16 (29.1)	4.87	1.02 to 26.81	0.0837
Male sex–no (%)	2 (33.3)	8 (14.5)	2.94	0.48 to 15.21	0.2526
Seropositive–no (%)	1 (16.7)	31 (56.4)	0.15	0.01 to 1.33	0.0933
Associated SjD	0 (0)	8 (14.5)	0	0 to 3.93	>0.9999
ILD	1 (16.7)	1 (1.8)	10.8	0.48 to 207.1	0.1885
Vasculitis	0 (0)	3 (5.5)	0	0 to 11.33	>0.9999
Other immune dysregulation (beyond arthritis)–no (%)	3 (50)	26 (47.3)	1.11	0.24 to 5.1	>0.9999
Suggestive family history‡–no (%)	6 (100)	14 (25.5)	+inf.	4.56 to inf.	0.0007 (***)
Difficult-to-treat–no (%)	1 (16.7)	12 (21.8)	0.72	0.06 to 5.16	>0.9999

* p<0.001.

† Prior to the age of 40 years.

‡ First-degree relative with autoimmune disease, chronic inflammatory disease (including rheumatic disease, inflammatory bowel disease) or immunodeficiency.

IBD, inflammatory bowel disease; IEI, inborn errors of immunity; ILD, interstitial lung disease; inf., infinity; N, total number; n.a., not applicable; no., number; RA, rheumatoid arthritis; SID, secondary immunodeficiency; SjD, Sjögren’s disease.

## Discussion

In this study, we identified rare germline variants in genes associated with IEI in patients with RA and immunodeficiency induced by treatment with DMARDs. These findings suggest a potential overlap in the genetic background of immunodeficiency in RA and monogenic IEIs, whose phenotypic spectrum, in addition to susceptibility to infections, includes manifestations of immune dysregulation that may mimic oligogenic or polygenic rheumatic disorders.

Among the identified variants, two could be classified as pathogenic, as their pathogenicity has been demonstrated in previous studies and may therefore establish the diagnosis of an IEI in the patients harbouring them. Although only a few among tested patients were identified to harbour definitely pathogenic variants, the identification of such variants had direct therapeutic implications. For example, the diagnosis of STAT3 gain-of-function syndrome should primarily prompt treatment with JAKi, whereas alternative DMARDs may further aggravate the disease-intrinsic immunodeficiency. In the case of the IκBα truncating variant, identification of an autoinflammatory, inflammasome-mediated mechanism of arthritis may explain the observed therapeutic efficacy of TCZ and led to consideration of interleukin-1 inhibition.^18^ In contrast, the clinical relevance of VUS requires cautious interpretation and additional functional or segregation studies before therapeutic decisions are made. Furthermore, establishing a genetic diagnosis enabled family screening, including relatives previously diagnosed with rheumatic disorders, thereby expanding the number of identified cases. The diagnosis of specific IEI can have implications for patient follow-up surveillance, given the risk of particular manifestations of immune dysregulation, such as enteropathies or cytopenias and malignancies. In addition, identification of an IEI may guide strategies to reduce infection risk. Besides skipping DMARDs and systemic glucocorticoids, management of high infection risk can include timely initiation of immunoglobulin replacement therapy, which in primary antibody deficiencies may be indicated even in cases of mild hypogammaglobulinaemia. Preventive measures may further include tailored vaccination strategies and antimicrobial prophylaxis targeting infections known to occur in specific IEIs, such as the introduction of antiviral agents to prevent herpes virus reactivation in patients with loss-of-function variants in *TICAM1*.

While patient complexity and the presence of a clinically evident primary immunodeficiency often prompt genetic testing for an underlying IEI, we have previously identified IEIs in a cohort of patients diagnosed with rheumatic disorders who also developed hypogammaglobulinaemia during treatment with diverse DMARDs.^17^ Furthermore, family screening in the context of studies identifying IEI-causing variants has revealed the segregation of deleterious variants in relatives with milder phenotypes, including a single well-classifiable rheumatic disorder.^45^,^46^ The aforementioned observations suggest that the current perception of IEIs as primarily disorders of complex autoimmunity or clinically apparent immunodeficiency may be biased. Such a bias may stem from the fact that patients with marked immunodeficiency are more likely to undergo genetic testing, whereas in most healthcare systems, genetic screening for IEIs is not routinely available for patients with single manifestations of immune dysregulation, including rheumatic disorders. Consistent with this, a study on SOCS1 insufficiency, a monogenic disorder with diverse manifestations of immune dysregulation, uniquely assessed the prevalence of an IEI within the category of diseases of immune dysregulation by analysing population-scale WES data from the UK Biobank, identifying individuals carrying pathogenic SOCS1 variants who were largely oligosymptomatic or asymptomatic.^47^ Compared with patients in the European Society for Immunodeficiencies registry, which collects cases from specialised IEI centres, carriers of pathogenic SOCS1 variants identified within the UK Biobank were more frequently diagnosed with rheumatic disorders. In line with the latter, the identification of IEI-causing variants among patients with RA in the present study, although rare, demonstrates that monogenic IEIs can present with rheumatic disease, which can be their first or dominant clinical manifestation.

Importantly, several IEIs caused by monoallelic variants, particularly disorders associated with *NFKB1*, *STAT3* and *CTLA4*, are characterised by incomplete penetrance and variable expressivity.^34 48 49^ Consequently, carriers may present with markedly heterogeneous phenotypes ranging from asymptomatic or oligosymptomatic disease to clinically overt immunodeficiency and immune dysregulation, while not all patients develop hypogammaglobulinaemia. The latter suggests the contribution of additional genetic modifiers, epigenetic regulation and environmental factors to disease expression. In this context, glucocorticoids and DMARDs may unmask subclinical immunodeficiency and precipitate the onset of clinically overt hypogammaglobulinaemia and infectious complications.

Despite the potential clinical relevance of identifying an underlying IEI, the broader implementation of genetic testing strategies in rheumatic diseases requires careful consideration. Several limitations currently represent obstacles to broad routine genetic screening, including the costs and availability of testing, the incomplete understanding of the natural history of several monogenic disorders, the variable penetrance and expressivity of IEI-associated variants and the still very limited availability of disease-specific targeted therapies or outcome data following genetic diagnosis. Nevertheless, genetic testing appears justified in selected patients with suggestive clinical features, including SID and a suggestive family history. In this context, a tiered diagnostic strategy may represent a pragmatic approach, with targeted IEI gene panels or focused genetic analyses guided by the patient’s clinical phenotype and family history as first-line approaches, while WES may be particularly valuable in unresolved or highly suspicious cases, given its ability to identify rare variants outside predefined panels and to accommodate the continuously expanding spectrum of IEI-associated genes.

Glucocorticoids and DMARDs used in the management of RA are recognised contributors to SID.^21^ Hypogammaglobulinaemia, defined as reduced serum IgG levels, occurs in a substantial subset of patients with RA, especially the subset of RTX-treated patients, where up to 20%–24% develop hypogammaglobulinaemia; baseline IgG levels and cumulative RTX exposure have been associated with the development of hypogammaglobulinaemia, whereas concomitant MTX therapy has been reported to mitigate this risk.^11 50^ Beyond RTX, hypogammaglobulinaemia has also been associated with glucocorticoid exposure and csDMARDs. In addition, glucocorticoid therapy increases the risk of infection in patients receiving TNFi in a dose-dependent manner.^9^ In the present study, SID was associated with an earlier diagnosis of RA and with seronegative disease. Consistent with previous reports, hypogammaglobulinaemia was more frequently observed among RTX-treated patients.

Our study has several limitations. Despite applying stringent variant filtering criteria, including rarity and in silico pathogenicity predictions, the identified variants were not systematically confirmed by Sanger sequencing and their functional relevance was not experimentally validated, while the majority of identified variants were classified as VUS. Furthermore, no segregation analyses were performed in families of patients carrying VUS, limiting the ability to assess the potential pathogenicity of the identified variants. In addition, the retrospective design of the study may have limited the accurate assessment of infection history and the impact of medications, particularly glucocorticoid dose. This limitation was partially addressed by classifying patients receiving prophylactic anti-infective therapy as having SID.

Beyond the direct implications for clinical management of patients with RA in the context of an underlying IEI, our findings suggest that unusual infectious complications or persisting hypogammaglobulinaemia perceived as secondary to DMARDs should prompt consideration of the diagnosis of an IEI by means of genetic testing. The latter may be relevant in case of trials evaluating the safety of immunomodulatory drugs to treat RA. In particular, rarely reported opportunistic infections in patients receiving DMARDs may not solely reflect drug-related adverse effects, but could instead be the consequence of a rare genotype.

## Supplementary material

10.1136/rmdopen-2026-007046 online supplemental file 1

## Data Availability

Data are available on reasonable request.
