# Supplementary material for: Rare variants in genes related to inborn errors of immunity in patients with rheumatoid arthritis and secondary immunodeficiency
Source: RMD Open. 2026 Jul 3;12(3):e007046. doi: 10.1136/rmdopen-2026-007046 (PMC13343069; doi:10.1136/rmdopen-2026-007046)
Supplement: online supplemental file 1 [file rmdopen-12-3-s001.docx]

**SUPPLEMENTAL MATERIAL**

**Suppl. text**: Genes related to disorders of tables 2 to six of the classification of IEI from the International Union of Immunological Societies Expert Committee (see reference [13]), evaluated for variants that could account for late-onset immunodeficiency by means of whole-exome sequencing in the present study:

*ACP5, AP3B1, APOL1, ARPC1B, ARPC5, B2M, BAFF-R, BCL10, BCL11B, BLNK, BTK, CARD11, CCBE1, CBLB, CCR2, CD19, CD21, CD247, CD25, CD274, CD3D, CD3E, CD3G, CD40, CD40LG, CD45, CD79A, CD79B, CD81, CD8A, CHUK, CIITA, CLCN7, COPG1, CORO1A, CR2, CSF3R, CTLA4, CTSC, CYBA, CYBB, CYBC1, DBF4, DCLRE1B, DEF6, DOCK11, DOCK2, DOCK8, DPP9, DUT, ELANE, EPG5, ERCC6L2, ERN1, FAT4, FCHO1, FERMT3, FLT3L, G6PC3, GATA2, GFI1, GIMAP6, GINS4, GTF3A, HAX1, HMOX, IFIH1, IFNAR1, IFNAR2, IFNGR1, IFNGR2, IGHM, IGLL1, IKBKB, IKBKE, IKZF1, IKZF2, IL12B, IL12RB1, IL12RB2, IL21, IL21R, IL27RA, IL2RA, IL2RB, IL6R, IL6ST, IRAK1, IRAK4, IRF1, IRF2BP2, IRF4, IRF7, IRF8, IRF9, ISG15, ITCH, ITGB2, ITK, ITPKB, ITPR3, JAGN1, JAK1, JAK3, KARS1, LACC1, LAT, LCK, LRBA, LRRK1, LY96, LYST, MALT1, MAN2B2, MAP3K14, MCTS1, MD2, MECOM, MSN, MYD88, NBAS, NCF1, NCF2, NCF4, NEMO, NFATC1, NFATC2, NFKB1, NFKB2, OAS1, OAS2, OSTM1, PAX1, PAX5, PEPD, PI4KA, PIK3CD, PIK3R1, PLCG1, PLEKHM1, POLD1, POLD2, POLD3, POLR3A, POLR3C, POLR3F, PRF1, PRIM1, PTCRA, PTEN, PTPN2, PTPRC, RAB27A, RAC2, RAD50, RANBP2, RBCK1, RECQL4, REL, RELA, RELB, RFX5, RFXANK, RFXAP, RHBDF2, RHOH, RIPK3, RNASEH2A, RNASEH2B, RNASEH2C, RNASEL, RNF31, RORC, RPSA, SAMHD1, SASH3, SEC61A1, SGPL1, SH2B3, SKIV2L, SLC19A1, SLC35C1, SLC39A7, SMAD3, SNX10, SOCS1, SP110, SPPL2A, SRP19, SRP72, SRPRA, STAT1, STAT2, STAT3, STAT6, STN1, STX11, STXBP2, TACI, TAP1, TAP2, TAPBP, TCF3, TCIRG1, TFRC, TICAM1, TIRAP, TLR3, TLR4, TLR7, TLR8, TNFRSF11A, TNFRSF13B, TNFRSF13C, TNFRSF4, TNFSF11, TNFSF9, TOP2B, TPP2, TRAC, TRAF3, TREX1, TRNT1, TTC37, TTC7A, TYK2, UNC13D, UNC93B1, USB1, USP18, VIPAS39, VPS13B, VPS33B, VPS45, WAS, WDR1, WIPF1, WIPF2, ZAP70, ZNF341, ZNSF1*.

Genes evaluated for variants that could account for immunodeficiency by means of targeted next-generation sequencing in our previous study (see reference [18]):

*ADA, AICDA, AKT1, BCL2L1, BCL6, BLNK, BTK, CASP8, CD19, CD27, CD274, CD28, CD40, CD40LG, CD79A, CD79B, CD80, CD81, CD86, CECR1, CLEC16A, CR2, CTLA4, CXCL12, CXCR4, CXCR5, DCLRE1C, DOCK8, FCGR3A, FCGRT, FOXP3, GATA2, GRB2, ICOS, ICOSLG, IGHM, IGLL1, IKBKB, IKBKE, IKBKG, IKZF1, IL10RA, IL10RB, IL17A, IL17RA, IL21, IL21R, IL4I1, IRF2BP2, IRF4, IRF8, LRBA, MCM10, MCM4, MICALL2, MLH1, MS4A1, MSH2, MSH5, MYO5B, NBN, NFKB1, NFKB2, NFKBIA, NLRP12, PDCD1, PDCD1LG2, PIK3AP1, PIK3C2A, PIK3CD, PIK3R1, PIK3R4, PRDM1, PRKCD, PRKD1, RAD50, RAG1, RAG2, REL, RELA, RELB, RIF1, RLTPR, RTEL1, SEC61A1, SEC61A2, SH2D1A, SH3KBP1, SOCS1, STAT1, STAT3, STAT4, TCF3, TNFRSF10A, TNFRSF13B, TNFRSF13C, TNFRSF17, TNFRSF18, TNFRSF4, TNFSF10, TNFSF13, TNFSF13B, USP8, VAV1*.

**Suppl. Table 1**: Factors associated with secondary immunodeficiency in multivariable logistic regression analysis

| **Characteristic** | **OR (95% CI)** | **95% CI** | ***p*-value**^1^ |
| --- | --- | --- | --- |
| Median age (IQR)-years | 1.04 | 1.01-1.07 | 0.00842 (**) |
| Age at diagnosis of RA (IQR)-years | 0.95 | 0.93-0.98 | 0.000127 (***) |
| Seropositive-no (%) | 0.21 | 0.11-0.38 | <0.0001 (****) |
| associated SjD | 1.68 | 0.65-4.32 | 0.284 |
| ILD | 0.84 | 0.21-3.32 | 0.809 |
| Vasculitis | 2.73 | 0.76-9.79 | 0.124 |
| MTX-no (%) | 1.31 | 0.71-2.43 | 0.383 |
| LFN-no (%) | 1.22 | 0.3-4.85 | 0.782 |
| SSZ-no (%) | 0.181 | 0.02-1.47 | 0.11 |
| RTX-no (%) | 2.36 | 1.07-5.21 | 0.0339 |
| TNFi-no (%) | 0.5 | 0.2-1.3 | 0.155 |
| IL-6Ri-no (%) | 0.7 | 0.16-2.98 | 0.63 |
| Abatacept-no (%) | 2.78 | 0.8-9.66 | 0.107 |
| JAKi-no (%) | 0.97 | 0.3-3.15 | 0.966 |
| bDMARD, biological disease-modifying antirheumatic drugs; csDMARDs, conventional synthetic disease-modifying antirheumatic drugs, GC, glucocorticoids; IL-6Ri, interleukin 6 receptor inhibitor; IQR, interquartile range; JAKi, Janus kinase inhibitors; LFN, leflunomide; MTX, methotrexate; N, total number; no, number; RA, rheumatoid arthritis; RTX, rituximab; SjD, Sjögren’s disease; SSZ, sulfasalazine; TNFi, tumor necrosis factor inhibitor; tsDMARDs, targeted synthetic disease-modifying antirheumatic drugs  ^1^p < 0.05 *; p < 0.01 **; p < 0.001 ***; p < 0.0001 **** | | | |

**Suppl. Table 2**: IgG level and immunophenotypic parameters of patients with monoallelic variants in genes associated with autosomal dominant IEIs

| Pat. ID | current*  IgG  (7-16 g/l) | IgG at first presentation  (7-16 g/l) | lymphocyte  count**  (1100-4500 cells/µl) | lymphocytes  %  (20-44% WBC) | CD3 %  (55-83% lymph.) | CD4 %  (28-57% lymph.) | CD8 %  (10-39% lymph.) | B cells  (6-19% lymph.) | Naïve B cells  (42.6-82.3% B cells) | Cl. sw. memory B cells  (6.5-29.1% B cells) | CD21^low^ B cells  (0.9-7.6% B cells) |
| --- | --- | --- | --- | --- | --- | --- | --- | --- | --- | --- | --- |
| 1 | 5.39 | 7.37 | 490 | 14 | 69 | 50.1 | 17.7 | 2 | n.m. | n.m. | n.m. |
| 3 | 6.38 | 7.44 | 2070 | 23 | 83 | 56.4 | 24.2 | 18 | 81.7 | 3.4 | 0.6 |
| 4 | 3.83 | 7.66 | 1224 | 12 | 86 | 33.5 | 52.5 | 0.1 | n.m. | n.m. | n.m. |
| 5 | 6.33 | 7.87 | 420 | 6 | 59 | 26 | 32.4 | 3 | 23 | 10 | 0.3 |
| 7 | 2.15 | 7.7 | 1914 | 29 | 56 | 23.9 | 30.2 | 20 | n.a. | n.a. | n.a. |
| 8 | 5.33 | 7.4 | 3234 | 33 | 61.4 | 31.3 | 26.1 | 14 | 71.6 | 9.8 | 1.4 |
| 20 | 3.35 | 9.6 | 1068 | 6 | 64.4 | 14.7 | 45.5 | 13 | 48.3 | 14.1 | 1.8 |
| 24 | 5.96 | 7.33 | 1550 | 9 | 75.3 | 34 | 38.5 | 0.6 | n.m. | n.m. | n.m. |
| 26 | 6.08 | 12.2 | 1512 | 24 | 83.4 | 40.4 | 34.8 | 0.7 | n.m. | n.m. | n.m. |
| 35 | 5 | 8.66 | 2185 | 23 | 60 | 15.7 | 39.7 | 5.3 | 23 | 22.3 | 26 |
| 38 | 5.26 | 7.09 | 1825 | 25 | 63.3 | 44.9 | 18.1 | 13 | 93 | 6.3 | 0 |
| 44 | 6.55 | 7.64 | 1692 | 36 | 51.3 | 40.5 | 11.5 | 13.9 | 30.8 | 1.2 | 3 |
| 49 | 4.14 | 7.09 | 605 | 11 | 52.4 | 45.6 | 6.8 | 11.1 | 75.8 | 6.9 | 5 |
| 68 | 6.29 | 8.61 | 667 | 9 | 35 | 11.3 | 11.2 | 18.1 | 18.1 | 3.1 | 4 |
| 69 | 5.23 | 12.2 | 2230 | 18 | n.a. | n.a. | n.a. | n.a. | n.a. | n.a. | n.a. |
| *at time of the study, reference ranges for each parameter are shown in parentheses  **after the diagnosis of secondary immunodeficiency or after the diagnosis of lymphopenia, matching the presented lymphocyte subset counts  ^#^documented previous treatment with immunomodulatory agents  Reference range for each parameter is shown in brackets. Values deviating from reference range are highlighted with bold characters  Cl.sw., class-switched; lymph., lymphocytes; n.a., not available; n.m., not measurable due to low total B cell count; Pat. ID, patient identification number; WBC, white blood cells | | | | | | | | | | | |

**Suppl. Table 3**: Sensitivity analysis

|  | **Current study** | **Current study excluding patients from previous study [18] and patients w/o persistent hypogammaglobulinemia** | **Previous study**  **[18]** | ***p*-value** |
| --- | --- | --- | --- | --- |
| Screened patients | 701 | 553 | 274 | n.a. |
| SID-no (% screened) | 70 (10) | 51 (9.2) | 18 (6.6) | 0.2296 |
| At least one VUS in AD-IEI-related gene-no (% SID) | 15 (21.4) | 12 (23.5) | 6 (33.3) | 0.5336 |
| At least one class 4 or 5 variant in AD-IEI-related gene-no (% SID) | 3 (4.3) | 3 (5.9) | 2 (11.1) | 0.5931 |
| AD-IEI, autosomal dominant inborn error of immunity; no., number; SID, secondary immunodeficiency; VUS, variant of uncertain significance; w/o, without | | | | |


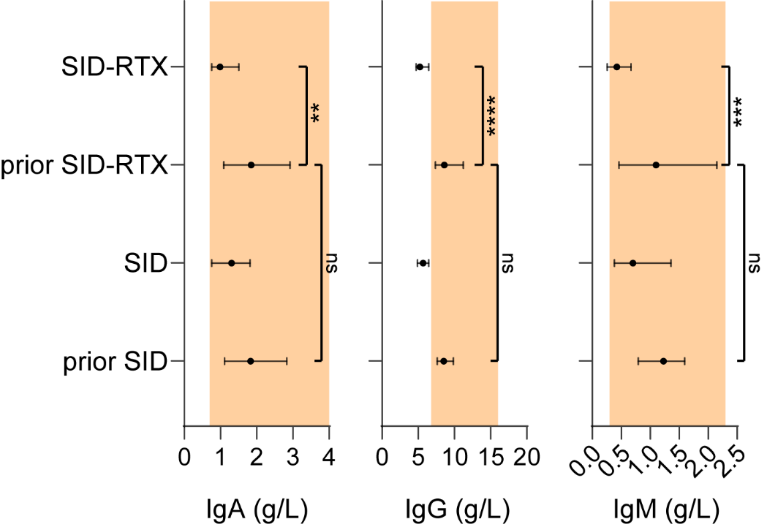


**Suppl. Figure 1:** Baseline serum immunoglobulin levels prior to rituximab (RTX) exposure in RA patients with SID. Serum IgA, IgG and IgM levels measured at first presentation prior to RTX treatment (prior SID-RTX) in RA patients who subsequently developed RTX-associated SID (SID-RTX) compared with the remaining SID subgroup (at first presentation, prior SID in comparison to SID). Data are presented as median and interquartile range (IQR). Shaded areas indicate the respective reference ranges (p < 0.01 **; p < 0.001 ***; p < 0.0001 ****).
